# Within- and Between-Individual Variations in Protein, Sodium, Potassium, and Phosphorus Intake Estimated from Urinary Biomarkers and Dietary Records in Individuals with Type 2 Diabetes Mellitus

**DOI:** 10.3390/nu17111757

**Published:** 2025-05-22

**Authors:** Tomoya Takaoka, Daiki Watanabe, Manami Hosokawa, Kana Hosokawa, Satoshi Kubota, Yuko Kawai, Fumi Oono, Yumiko Inoue, Chieko Zakoji, Ako Oiwa, Ai Sato, Masanori Yamazaki, Mitsuhisa Komatsu

**Affiliations:** 1 Medical Science Division, Department of Medical Science, Graduate School of Medicine, Science and Technology, Shinshu University, 3-1-1 Asahi, Matsumoto City 390-8621, Nagano, Japan; ttakaoka@shinshu-u.ac.jp; 2 Division of Clinical Nutrition, Shinshu University Hospital, 3-1-1 Asahi, Matsumoto City 390-8621, Nagano, Japan; 3 Faculty of Sport Sciences, Waseda University, 2-579-15 Mikajima, Tokorozawa City 359-1192, Saitama, Japan; d2watanabe@aoni.waseda.jp; 4 National Institute of Health and Nutrition, National Institutes of Biomedical Innovation, Health and Nutrition, 3-17 Senrioka-Shimmachi, Settsu City 566-0002, Osaka, Japan; 5 Department of Diabetes, Endocrinology and Metabolism, Division of Internal Medicine, School of Medicine, Shinshu University, 3-1-1 Asahi, Matsumoto City 390-8621, Nagano, Japan; 6 Department of Epidemiology and Preventive Medicine, Graduate School of Medicine, Gifu University, 1-1 Yanagido, Gifu City 501-1193, Gifu, Japan; 7 Department of Social and Preventive Epidemiology, Division of Health Sciences and Nursing, Graduate School of Medicine, The University of Tokyo, 7-3-1 Hongo, Bunkyo-Ku 113-8654, Tokyo, Japan; 8 Graduate School of Sport Sciences, Waseda University, 2-579-15 Mikajima, Tokorozawa City 359-1192, Saitama, Japan; 9 Center of Diabetes, Okaya City Hospital, 4-11-33 Honcho, Okaya City 394-8512, Nagano, Japan

**Keywords:** variation, dietary assessment, urinary biomarker, dietary record, type 2 diabetes

## Abstract

Background/Aim: Appropriate dietary assessment plays a crucial role in individualized nutritional therapy for individuals with type 2 diabetes mellitus (T2DM). Daily dietary variations must be considered in the estimation of usual dietary intake, and such data are limited in individuals with T2DM. This study aimed to evaluate within- and between-individual variations in protein, sodium, potassium, and phosphorus intakes estimated from 24 h urine collection (24 h UC) and semi-weighted dietary records (DRs) in Japanese individuals with T2DM. Methods: This study included 39 Japanese individuals (26 males, 13 females; mean age 64.6 years) with T2DM who attended two hospitals. Protein, sodium, potassium, and phosphorus intakes were estimated using 2-day 24 h UC and 3-day DRs and within- and between-individual variations were calculated using a one-way analysis of variance. Results: The mean protein, potassium, and phosphorus intakes did not significantly differ between 24 h UC and DRs. However, sodium intake was lower when estimated by DRs than by 24 h UC. The coefficients of within-individual variation (CV_w_) differed between 24 h UC and DRs. For protein and phosphorus, the CV_w_ values were smaller by 12.5% and 8.0% in males and 2.3% and 3.0% in females, respectively, for 24 h UC than DRs. For sodium and potassium, the CV_w_ values were smaller by 7.0% and 4.8% in males, but larger by 5.0% and 3.3% in females, respectively, for 24 h UC than DRs. Conclusions: Our findings demonstrated that 24 h UC showed smaller within-individual variations than DRs for protein and phosphorus in both sexes, with sex-specific differences for sodium and potassium.

## 1. Introduction

Nutrition therapy plays a crucial role in the management of type 2 diabetes mellitus (T2DM), such as maintaining appropriate blood glucose levels, maintaining appropriate body weight and reducing obesity rates, and delaying or preventing complications [1,2]. Previous randomized clinical trials have revealed a significant reduction in HbA1c levels with individualized nutritional therapy compared to usual diabetes care [3,4]. Therefore, appropriate dietary assessment methods are fundamental to accurate dietary intake estimation and effective nutrition therapy. The accuracy of dietary assessment results is crucial not only for individualized nutrition therapy but also for identifying food intake patterns and understanding diet–disease relationships in nutritional epidemiology [5,6].

Dietary surveys commonly use self-reported dietary assessment methods, such as dietary records (DRs), 24 h dietary recalls, and food frequency questionnaires [7,8,9,10]. However, these methods are prone to measurement errors due to daily dietary variation and their reliance on memory and recording eating behaviors [9,10,11], which can distort the estimated relationships between dietary factors and the risk of T2DM [6]. As such, objective biomarkers, such as 24 h urine collection (24 h UC), are considered the gold standard for estimating protein, sodium, and potassium intakes as they are not dependent on self-reporting [9]. Research has shown that higher sodium (salt) intake, as estimated by 24 h UC, is associated with increased mortality in individuals with T2DM [12]. However, while 24 h UC is a valuable assessment tool, it is still affected by daily dietary variations, similar to self-reported methods [13]. Thus, the estimation of usual dietary intake should account for daily dietary variations to improve its accuracy, regardless of the assessment method used.

Several studies have investigated the number of days and the group size required to accurately estimate usual dietary intake, considering within- and between-individual daily dietary variations measured by 24 h UC [14,15] and DRs [15,16,17,18]. Notably, within-individual variations are typically smaller with 24 h UC than other methods such as DRs [13,19,20], which may be due to the low risk of reporting bias with 24 h UC. Therefore, fewer days may be needed to estimate individual usual mean intakes using 24 h UC than DRs. Studies employing both estimation methods simultaneously in a single cohort are necessary to validate this hypothesis, as participant characteristics may play a critical role. However, such studies are few in number, and these investigations are typically limited to healthy populations [19,20]. To the best of our knowledge, no studies have specifically examined the extent of within- and between-individual daily dietary variations or compared these variations between 24 h UC and DRs in individuals with T2DM. Clarifying the extent of within- and between-individual variations in patients with T2DM is crucial for improving the accuracy of dietary assessment in individuals and populations and developing effective individualized nutrition therapy for T2DM. The aim of this study was therefore to compare within- and between-individual variations between 24 h UC and DRs estimations of dietary intake of protein, sodium, potassium, and phosphorus in Japanese individuals with T2DM. We also aimed to determine the required number of measurement days and group sizes for both 24 h UC and DRs based on these variations. We hypothesized that 24 h UC would demonstrate smaller within-individual variance and larger between-individual variance than DRs because 24 h UC has a low risk of reporting bias and thus reduces measurement errors.

## 2. Materials and Methods

### 2.1. Study Population

This cross-sectional study recruited 40 Japanese individuals with T2DM who attended the Department of Diabetes and Endocrinology of Shinshu University Hospital or the Diabetes Center at Okaya City Hospital (located in Matsumoto and Okaya in Nagano prefecture) between July 2023 and September 2024. The inclusion criteria were as follows: (1) Japanese females and males aged 30–79 years and (2) diagnosed with T2DM based on the diagnostic criteria established by the Japan Diabetes Society [21]. The exclusion criteria were (1) a history of renal disease (diabetic nephropathy stage 3 or higher), severe cardiovascular disease, or cancer treatment within the past 5 years; (2) cognitive impairments; (3) conditions with significant metabolic effects, such as endocrine and metabolic disorders (e.g., uncontrolled Graves’ disease); (4) use of loop diuretics; (5) use of diuretics other than loop diuretics for <3 months; (6) use of sodium–glucose co-transporter-2 (SGLT2) inhibitors for <3 months; or (7) significant body weight changes (>5%) within the past 6 months. A total of 40 individuals (26 males and 14 females) agreed to participate in the study.

Of these participants, we excluded those who had one or more abnormal 24 h UC measurements (e.g., urinary creatinine <10 mg/kg body weight/day for males or <7.5 mg/kg body weight/day for females [22,23,24], or urine volume ≤ 500 mL in 24 h) or reported implausible estimated energy intakes (<500 or >5000 kcal/day) by DRs. One woman was excluded due to an abnormal 24 h UC measurement, but no participants were excluded due to implausible estimated energy intakes. Ultimately, this study included 39 individuals aged 42–76 years (26 males and 13 females).

This study protocol was approved by the Ethics Committee of Shinshu University School of Medicine (approval number: 5844; date of approval: 15 May 2023). The study rigorously adhered to the ethical principles outlined in the Declaration of Helsinki. Prior to enrollment, prospective participants received explanations of the study purpose and design from study dietitians and provided written informed consent. Our research is reported using the STROBE-nut checklist [25].

### 2.2. Measurement Schedule

**Figure 1:**
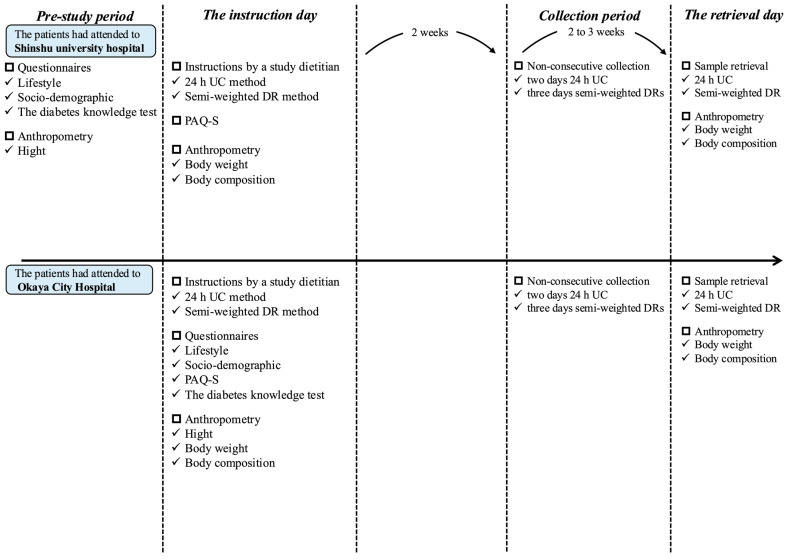
Study schedule; 24 h UC, 24 h urine collection; DR, dietary record; PAQ-S, physical activity questionnaire—short.

This study utilized data collected over 2 or 3 days between September 2023 and October 2024 (Figure 1). To standardize the data collection procedure, the study dietitians shared a detailed manual of measurement procedures in advance. The measurement methods used were 24 h UC (two non-consecutive days; weekdays and/or weekend days), semi-weighted DRs (three non-consecutive days; two weekdays and one weekend day), questionnaires, and anthropometric measurements (body height and weight). On the instruction day for 24 h UC and DR, each participant received detailed guidance for both procedures. The measurement schedule was arranged according to individuals’ schedules, with completion planned for 15–35 days after the instruction day; 24 h UC and semi-weighted DR assessments were performed on either the same or different days, and participants were contacted to confirm the procedure 1 day prior (9 individuals completed two-day 24 h UC and DR measurements on the same days; 18 individuals completed one-day 24 h UC and DR measurements on the same day; and 12 individuals did not complete 24 h UC and DR measurements on the same day).

### 2.3. Twenty-Four-Hour Urine Collection

Two non-consecutive 24 h UC measurements were conducted to determine the 24 h urine volume and urinary creatine, phosphorus, urea nitrogen, sodium, and potassium concentrations. The individuals were provided with one 3 L plastic bottle, four 1 L plastic bottles, two 250 mL plastic bottles, and twenty 400 mL cups. The study dietitians (TT, KH) provided participants with both written and verbal instructions for urine sample collection, using illustrated figures as examples. On the day of collection, participants discarded their first urine specimen and then collected all specimens thereafter. The final urine specimen was collected on the following day at the same time that the first specimen had been discarded the previous day. After all specimens were collected, individuals measured the total urine volume using a digital scale (KS-832, Dretec, Saitama, Japan; ±2 g precision for 0–750 g and ±3 g precision for 750–3000 g). Urine was assumed to be approximately 1.0 g/mL [26]. The well-stoppered 3 L or 1 L bottle was shaken approximately ten times, and an aliquot of pooled urine was placed into a 250 mL plastic bottle, which was then preserved in the participant’s freezer. The participants recorded the start time, finish time, and total urine volume as well as the estimated volume of any urine they forgot or failed to collect. We collected the 250 mL bottles containing urine specimens and the recording sheets. Each urine sample was preserved at −80 °C until analysis. The 24 h urine volume was adjusted based on the recording sheet to account for estimated miscollected urine volume and the collection time [27]: adjusted 24 h urine volume (mL/day) = (24 h urine volume [mL] + miscollected urine volume [mL])/collection hours (finish time—start time) × 24 h. Miscollected urine volume was estimated from reported urine that was forgotten or failed to be collected. The collected urine specimen was transferred to LSI Medience Corporation (Saitama, Japan), and urinary creatine, phosphorus, urea nitrogen, sodium, and potassium concentrations were measured. Creatinine and phosphorus concentrations were measured using the enzyme test (the L-Type Wako CRE·M, the DeterminerL IP II assay), urea nitrogen using the urease and leucine dehydrogenase method (the L-Type Wako UN·V assay), and sodium and potassium using the electrode method (A JCA-BM8060 clinical chemistry analyzer, JEOL Limited, Tokyo, Japan). The 24 h excretion volume was calculated by multiplying the measured concentration by the adjusted 24 h urine volume. Protein intake was calculated based on urinary urea nitrogen (g/day) by multiplying by 9.08. This calculation assumes that urea nitrogen accounts for 85% of total urinary nitrogen [28], 81% of ingested nitrogen is excreted in urine [29], and nitrogen constitutes 16% of protein [29]. Sodium intake was calculated based on urinary sodium (mmol/day) by dividing by 0.86, assuming that 86% of ingested sodium was excreted through the urine [30]. Potassium intake was calculated based on urinary potassium (mmol/day) by dividing by 0.77, assuming that 77% of ingested potassium was excreted through the urine [31]. Urinary sodium (mmol/day) was multiplied by 23, assuming that 1 mmol of sodium was equal to approximately 23 mg of sodium and converting the measurement to sodium (mg/day). Similarly, urinary potassium (mmol/day) was multiplied by 39.1, assuming that 1 mmol of potassium was equal to approximately 39.1 mg of potassium and converting it to potassium (mg/day). Phosphorus intake was calculated based on urinary phosphorus (mg/day) by dividing by 0.627, assuming that 62.7% of ingested phosphorus was excreted through the urine [32].

### 2.4. Semi-Weighted Dietary Records

The semi-weighted DRs were collected on three non-consecutive days (two weekdays [Monday to Friday, except for national holidays] and one weekend day [Saturday, Sunday, or national holidays]). Blank recording sheets and a digital scale (KS-732, Dretec, Saitama, Japan; ±2 g precision for 0–500 g and ±3 g precision for 500–2000 g) were provided to each participant. The study dietitians (TT, KH) provided participants with both written and verbal instructions for keeping DRs, using completed recording sheets as examples. Participants were instructed to document and weigh all consumed foods and drinks, both inside and outside of their homes, on each recording day. On occasions when recording was difficult (e.g., dining out), participants were instructed to record as much information as possible, including the name of the menu item and restaurant, the consumed portion size (based on typical household measures), and details of the leftover details.

After completing the survey, participants submitted the recording sheets used on each survey day directly to the study dietitian after the survey was completed. The dietitian then reviewed the form and interviewed the participant directly, either in person or by phone or email, to address any missing or unclear information.

All collected records were reviewed, portion sizes were converted from estimated household measures to grams, and individual food, beverage, or seasoning items were coded by research dietitians (TT, KH) based on the 2015 version of the Standard Tables of Food Composition in Japan (STFC-J) [33]. Any foods, beverages, or seasonings that are not included in the STFC-J were assigned codes for similar items.

### 2.5. Other Measurements

Body height and weight were measured to the nearest 0.1 cm and 0.1 kg, respectively, while the participants were wearing light clothes and no shoes using a wall-mounted stadiometer (AD-6228A, A & D Medical, Tokyo, Japan) and body composition monitor (MC-780A-N, TANITA, Tokyo, Japan). Body mass index (BMI) was calculated as body weight (kg) divided by the square of body height (m). Socioeconomic status (e.g., living status, education, and household income), physical activity, cognitive function, and diabetes knowledge were assessed using a self-administered questionnaire, the Physical Activity Questionnaire—Short [34], and the Japanese version of the diabetes knowledge test [35], respectively. Laboratory parameters, including glycated hemoglobin, fasting plasma glucose, and estimated glomerular filtration rate were extracted from medical records.

### 2.6. Statistical Analysis

Individual characteristics are presented as means ± standard deviations (SDs) for continuous variables and as numbers (percentages) for categorical variables. We used untransformed data for protein, sodium, potassium, and phosphorus intakes estimated by 24 h UC and DRs because the estimated relative contributions of sources of variation were not significantly affected by a logarithmic transformation. Moreover, neither logarithmic nor Box–Cox transformations improved the assumption of homoscedasticity across covariates in the models [36]. Additionally, estimates derived from transformed nutrient data were difficult to interpret meaningfully, and back-transformations introduced bias in variance estimation [37].

The within-individual variance (σ_w_^2^) and between-individual variance (σ_b_^2^) for urinary excretion or dietary intake were calculated by one-way analysis of variance, with the factor representing between-individual variance and the residual representing within-individual variance. A correlation analysis was performed using Spearman’s correlation coefficient to assess the relationship between nutrient intakes measured by 24 h UC and DRs.

The group size (G) needed to estimate usual (“true”) group mean urinary excretion or dietary intake within a 95% CI within the specific percentage of deviation (D_0_) for 24 h UC or DRs was calculated using the following equation [38]: G = 1.96^2^ × [(CV_b_^2^ + CV_w_^2^)/D_0_^2^], where CV_b_ is the between-individual coefficient of variation, CV_w_ is the within-individual coefficient of variation, and D_0_ is a specific percentage (2.5, 5.0, 10.0, or 20.0%).

The number of days (N_R_) needed to ensure a specific correlation coefficient (r) between the observed and unobserved usual (“true”) mean urinary excretion or dietary intake in individuals was calculated using the following equation [39]: N_R_ = [r^2^/(1 − r^2^)] × VR, where VR is the variance ratio as determined by σ_w_^2^/σ_b_^2^. In this analysis, r serves as a measure of confidence regarding the ranking of individuals in a population using a specific correlation coefficient (0.75, 0.80, 0.90, or 0.95).

The number of days (N_I_) needed to estimate the usual (“true”) individual mean urinary excretion or dietary intake within a 95% CI within the specific percentage of deviation (D_1_) for 24 h UC or DRs was calculated using the following equation [38]: N_I_ = (1.96 × CV_w_/D_1_)^2^, where D_1_ is a specific percentage (5.0, 10.0, 20.0, or 30.0%).

Significance was set as a two-tailed *p* value of <0.05. All statistical analyses were performed using R statistical software version 4.3.3 (R Foundation for Statistical Computing, Indianapolis, IN, USA).

To confirm the urinary excretion rate of protein, sodium, potassium, and phosphorus, the crude urinary mean excretion of these nutrients was divided by the respective dietary mean intake estimated from DRs. To confirm the robustness of the study results, a sensitivity analysis was conducted using the following criteria: individuals with incomplete 24 h UC were excluded based on the criteria used in a previous healthy Japanese population study [40]. The ratio of observed to expected creatinine excretion was calculated using the equations proposed by Joossens et al. [27,41]. If the calculated ratio for a collection was <0.6 of the expected value, the UC was considered incomplete. Stratified analyses were conducted for insulin use (yes or no), SGLT2 inhibitor use (yes or no), and antihypertensive agent use (yes or no)—considered a reasonable proxy for treated hypertension and, thus, an indicator of hypertension history—and household income levels (<2 million JPY/year, 2–6 million JPY/year, ≥6 million JPY/year, or unknown).

In addition to the main analysis, a correlation analysis was performed to assess the relationship between inorganic phosphorus intake estimated by 24 h UC and phosphorus intake estimated by DRs. To estimate inorganic phosphorus intake, the urinary phosphorus-to-nitrogen excretion ratio (UP/UN, mg/g) was calculated, serving as a marker of inorganic phosphorus intake [42]. Additionally, Spearman’s correlation analysis was conducted between the UP/UN ratio and phosphorus intake estimated by DRs, which may better reflect dietary phosphorus intake than total urinary phosphorus excretion alone [42].

## 3. Results

### 3.1. Characteristics of the Study Participants

Table 1 presents the characteristics of the study participants. The mean age, BMI, and energy intake estimated by DRs were 64.6 years, 25.3 kg/m^2^, and 2061 kcal/day for all individuals; 66.2 years, 24.9 kg/m^2^, and 1760 kcal/day for females; and 63.8 years, 25.5 kg/m^2^, and 2211 kcal/day for males. Regarding diabetes treatment, 84.6% of all individuals (76.9% of females and 88.5% of males) used oral hypoglycemic agents and 48.7% (46.2% of females and 50.0% of males) used insulin.

### 3.2. Mean Nutrient Intake and Within-to-Between-Individual Variance Ratios Estimated Using 24 h UC and DRs

Table 2 shows the mean, SD, CV_w_, CV_b_, and VR values for protein, sodium, potassium, and phosphorus intakes as estimated by 24 h UC and DRs. The mean protein, potassium, and phosphorus intakes were comparable between 24 h UC and DRs in both sexes. In contrast, DR estimates were significantly lower than 24 h UC measurements of sodium intake in males (16.3% difference), although the discrepancy was non-significant in females (11.0% difference). The estimated excretion rates using the assumed excretion rates described above yielded similar findings, with the exception of sodium (Table S1).

As shown in Table 2, the CV_w_ values for protein and phosphorus were smaller when measured by 24 h UC than by DRs in both sexes (12.5 and 8.0% smaller in males and 2.3 and 3.0% smaller in females, respectively). The CV_w_ values for sodium and potassium were smaller in males (7.0 and 4.8% smaller, respectively) but larger in females (5.0 and 3.3% larger, respectively) when measured by 24 h UC than by DRs. For CV_b_, the protein values were larger in females (13.6% larger) but smaller in males (4.6% smaller) when measured by 24 h UC than by DRs. For sodium, the CV_b_ was larger in males (8.7% larger) but smaller in females (9.4% smaller) when measured by 24 h UC than by DRs. The CV_b_ for potassium was smaller when measured by 24 h UC than by DRs in both sexes (0.3 and 2.2% smaller in males and females, respectively). In contrast, the CV_b_ for phosphorus was larger when measured by 24 h UC than by DRs in both sexes (5.9 and 14.1% larger in males and females, respectively). The VRs were <1.0 for all nutrients estimated by both 24 h UC and DRs. The VRs for protein and phosphorus were smaller when measured by 24 h UC than by DRs in both sexes (0.34 and 0.19% smaller in males and 0.25 and 0.26% smaller in females, respectively), whereas the VRs for sodium and potassium were smaller in males (0.21 and 0.07% smaller, respectively) but larger in females (0.29 and 0.08% larger, respectively) when measured by 24 h UC than by DRs. The Spearman correlation coefficients between 24 h UC and DR estimates were as follows: protein: 0.39 (males) and 0.83 (females); sodium: 0.58 (males) and 0.48 (females); potassium: 0.58 (males) and 0.75 (females); and phosphorus: 0.33 (males) and 0.49 (females). Sensitivity analyses indicated that CV_w_ values were consistently smaller when measured by 24 h UC than by DRs across all nutrients (Table S2). Stratified analyses based on insulin, SGLT2 inhibitor, antihypertensive agent use, and household income exhibited similar results (Table S3). Spearman’s correlation between the UP/UN ratio and phosphorus intake estimated by DRs was not statistically significant in either sex, which was consistent with the correlation observed between 24 h UC and DRs (Table S4).

### 3.3. Required Group Size for Estimating Mean Nutrient Intake Using 24 h UC and DRs

Table 3 shows the group sizes required to estimate the usual mean intake of protein, sodium, potassium, and phosphorus with 95% CIs within a specified deviation from the group’s usual mean intake, as estimated by 24 h UC and DRs. The group size required to estimate the mean protein intake was smaller for 24 h UC than for DRs in males, but not in females. For sodium intake, the required group size was larger for 24 h UC than for DRs in males, but not in females. For potassium intake, the required group size was smaller for 24 h UC than for DRs in both sexes. In contrast, the required group size for phosphorus intake was larger for 24 h UC than for DRs in both sexes.

### 3.4. Days Required to Achieve a Specific Level of Correlation Between Observed and Unobserved Usual Nutrient Intake Using 24 h UC and DRs

Table 4 shows the number of days required to achieve a specific level of correlation between observed and unobserved usual mean intake of protein, sodium, potassium, and phosphorus, as estimated by 24 h UC and DRs. The number of days required to rank individuals within a group by protein and phosphorus intakes was shorter for 24 h UC than for DRs in both sexes. For sodium intake, the required number of days was shorter for 24 h UC than for DRs in males, but not in females. For potassium intake, the required number of days was similar for 24 h UC and DRs in both sexes.

### 3.5. Required Days for Estimating Mean Nutrient Intake Using 24 h UC and DRs

Table 5 shows the number of days required to estimate the usual mean intake of protein, sodium, potassium, and phosphorus with 95% CIs within a specified deviation from an individual’s usual mean intake, as estimated by 24 h UC and DRs. Fewer days were required to estimate the mean protein and phosphorus intakes with 24 h UC than with DRs in both sexes. For sodium and potassium intakes, fewer days were required for 24 h UC estimation than for DR estimation in males, but not in females.

## 4. Discussion

We evaluated within- and between-individual variations in protein, sodium, potassium, and phosphorus intakes as measured by 24 h UC and DRs in Japanese individuals with T2DM. The CV_w_ values and VRs were generally smaller for 24 h UC estimations than DR estimations. The CV_b_ values may show sex-specific differences across nutrients. The VRs were <1.0 for all nutrients estimated by both 24 h UC and DR. To our knowledge, this study is the first to assess within- and between-individual variations in dietary intake using simultaneous measurements from 24 h UC and DRs in individuals with T2DM.

The mean intakes of protein, potassium, and phosphorus did not differ significantly between 24 h UC and DRs, whereas sodium intake was significantly lower when measured by DRs than by 24 h UC. Consistent with our findings, a previous meta-analysis reported that 24 h dietary recalls underestimated mean sodium intake by an average of 607 mg/day compared with the 24 h UC-calculated values [43]. In Japan, approximately 60% of sodium intake is consumed through seasonings, particularly salt, soy sauce, and miso [44]. Therefore, sodium intake is more difficult to measure than protein, potassium, and phosphorus intakes using DRs due to measurement errors (difficulty in precisely measuring) in seasoning quantities. When 24 h UC is not feasible, considering the burden of DRs on individuals, multiple spot urine collections may be more useful than DRs for estimating usual sodium intake [40].

Our study found smaller total cohort CV_w_ values for protein (15.6 vs. 25.4%), sodium (24.0 vs. 27.9%), potassium (17.5 vs. 20.0%), and phosphorus (17.4 vs. 24.1%) intakes when measured by 24 h UC than by DRs. With regards to DR estimation, previous studies in Japanese populations have reported CV_w_ values of 18–31% for protein, 28–36% for sodium, 20–27% for potassium, and 19–28% for phosphorus [15,16,17,18,45,46,47,48] (see Table S5), which are similar to our results. Among prior studies, findings on the comparison between 24 h UC and DR estimations have been inconsistent. Suzuki et al. reported similar variations in sodium and potassium intakes between 24 h UC and DRs in a healthy Japanese population (mean age: 59 years for males, 58 years for females) [15]. In contrast, a UK study involving 13 healthy participants (mean age: 43 years) indicated varying CV_w_ values for protein and potassium intakes between 24 h UC and DR estimations (14.5 vs. 24.8% for protein and 19.0 vs. 17.9% for potassium, respectively) [31], which is similar to our results. These discrepancies may be due to differences in participant health status and age or study timing and location. The VR values estimated by DRs in our study were smaller than those of previous studies conducted in Japanese populations [15,16,17,18,45,46,47,48] (see Table S5). For example, Fukumoto et al. examined dietary intake using 16-day DRs and found VRs of 3.08 and 2.67, 4.67 and 5.35, 2.46 and 2.03, and 1.98 and 2.10 for protein, sodium, potassium, and phosphorus in healthy older females (aged 50–69 years) and males (aged 50–76 years), respectively [16]. In contrast, our results showed VRs of <1.0 for all nutrients estimated by both methods. In line with this, Watanabe et al. reported VRs of 0.81 and 0.87 for protein and 0.52 and 0.62 for sodium intake estimated by 2-day 24 h UC in females and males with chronic kidney disease, respectively [14]. These findings suggest that populations undergoing nutritional therapy, such as individuals with T2DM and chronic kidney disease, may have smaller VR values than healthy populations.

It is possible individuals with T2DM might maintain more consistent daily eating patterns than the general population in order to control blood glucose levels, which may result in smaller variance in daily dietary intake. Our participants exhibited relatively good blood glucose control (mean HbA1c levels 7.0%), which may suggest adherence to stable dietary intake. However, 24 h UC and DRs estimated their mean sodium intakes to be 5315 and 4530 mg/day, respectively, substantially higher than recommendations (<2300 mg/day) [1,2]. This indicates that while daily dietary variation may be stable, adherence to nutritional therapy may be suboptimal. Additionally, the observed CV_w_ values were generally comparable to those reported previously in studies of the general Japanese population, suggesting that the within-individual variation among individuals with T2DM may be comparable to that of the general population. Meanwhile, the between-individual variation was larger than the within-individual variation, reflecting considerable differences in dietary habits and intake levels among individuals with T2DM.

In our study, a smaller group size was required for 24 h UC than for DRs for assessing usual protein and potassium intakes. For example, 79 participants were required for the DR estimation of the group mean protein intake within 10% deviation, but only 68 participants were required for the 24 h UC estimation. Similarly, fewer days were required to assess individuals’ usual protein, sodium, potassium, and phosphorus intakes when using 24 h UC than when using DRs. For example, 7 days were required for the DR estimation of individual protein intake within 20% deviation, whereas only 3 days were required for the 24 h UC estimation. Our results pertaining to DRs were comparable with those of previous studies in Japan regarding required sample sizes and days needed for individual nutrition intake assessment [15,16,17,18,45,46,47]. Regarding 24 h UC, Bingham et al. showed that 8 days of collection were needed to estimate an individual’s usual protein intake within 10% standard error [29]. Our finding that only 10 days were needed to estimate usual individual mean protein intake within 10% deviation aligns with this report. Our results also showed that protein and potassium intake estimates did not significantly differ between 24 h UC and DRs, with acceptable to good ranking ability [49] (correlation coefficients: 0.39–0.83 for protein and 0.58–0.75 for potassium). Although DRs required more days and larger group sizes than 24 h UC, they remain a useful tool for estimating usual protein and potassium intakes in individuals with T2DM.

The main strength of this study is the simultaneous implementation of DR and 24 h UC measurements within a single cohort, using a detailed manual of measurement procedures from previous studies for quality control [40,50]. This minimized measurement bias. However, this study had some limitations. First, the participants with T2DM were recruited solely from two hospitals in rural areas of Japan. Thus, the sample was not randomly selected, which may have introduced a sampling bias, and was not nationally representative, which may limit generalizability. Moreover, these individuals may be more health-conscious than the general individuals with T2DM. Thus, caution should be exercised when adapting the results of this study to other populations. The Nagano Prefecture Health and Nutrition Survey is a cross-sectional household examination survey of healthy and clinical populations consisting of participants aged ≥18 years living in Nagano Prefecture. The survey is conducted every 3 years between late September and late November, with results available for 2010 to 2022 showing VRs for protein, sodium (calculated as salt [g/day]/2.54 × 1000), potassium, and phosphorus intakes based on 2-day semi-weighed household DRs in sub-samples [51,52,53,54,55] (Table S6). Additionally, the estimated usual mean intakes were reported by adopting the best-power method [56] to a one-day semi-weighted household DR in total individuals. Although these VRs varied between study years, making results comparison difficult, the weighted mean intakes were relatively close to our findings. This suggests that our study cohort may have similar dietary intake patterns to others living in Nagano Prefecture.

Second, the sample size was limited, particularly for females (*n* = 13), reducing the robustness and generalizability of the findings. However, sensitivity analyses demonstrated similar results in within and individual variations, suggesting that the small sample size may not significantly affect the directionality of the main findings. Meanwhile, mean differences and Spearman correlation coefficients between 24 h UC and DRs might differ by sex, insulin use, SGLT2 inhibitor use, antihypertensive agent use, or household income levels due to the small sample size. To estimate the group mean sodium intake within 10% deviation, 138 and 124 participants are needed for 24 h UC and DRs, respectively.

Third, although the results were analyzed according to sex, a separate analysis according to age was not performed due to the small sample size. Additionally, the cohort was typically older (mean age: 64.6 years), which may impact the VRs compared to young cohorts [16].

Fourth, self-reported methods are limited by within- and between-individual variation, as well as under- or over-reporting due to varying individual characteristics (e.g., age, weight status, smoking status, or living status) [57,58].

Finally, recruiting participants throughout the year may have introduced seasonal variations to the results. In the Japanese population, dietary intake, particularly fruits and vegetables that contribute to potassium intake, varies across seasons [59]. Consequently, 24 h UC and DR results might reflect these changes. Future studies should include larger, nationally representative samples with year-round measurements to facilitate analysis of seasonal variations.

Nevertheless, the findings of this study provide valuable information for planning dietary assessment and interpreting previously reported protein, sodium, potassium, and phosphorus intakes in individuals with T2DM. These findings assist in determining appropriate dietary assessment methods, required group sizes, and optimal assessment days for clinical settings and future nutrition research in T2DM populations. Due to the challenges associated with 24 h UC and DRs, it is essential to develop more efficient methods to accurately capture usual dietary intake. Methods developed based on the findings of the present study could improve individualized nutritional therapy for T2DM by offering more practical assessment tools.

## 5. Conclusions

Our findings in Japanese individuals with T2DM suggest that 24 h UC had smaller CV_w_ values than DRs for protein and phosphorus intakes in both sexes, with differences of 2.3–12.5%; for sodium and potassium, these values were smaller in males but larger in females. The within-individual variance was smaller than the between-individual variance for four nutrients estimated by both 24 h UC and DRs in both sexes. The number of days needed to estimate an individual’s mean intake, and the group size required to estimate the group mean intake differed by the assessment method (24 h UC or DRs) and sex. These differences in daily dietary variations between assessment methods and sexes should be considered when planning dietary assessments in individuals with T2DM. However, our study was limited to a small sample from a rural area in Japan. Future studies are warranted to examine these variations in a large nationwide sample of individuals with T2DM.

## Figures and Tables

**Table 1:** Baseline characteristics of the study participants.

	Total	Females	Males
	(*n* = 39)	(*n* = 13)	(*n* = 26)
Age, years	64.6 (8.4)	66.2 (5.8)	63.8 (9.4)
Height, cm	163.6 (9.4)	154.8 (8.5)	168.1 (6.2)
Body weight, kg	68.3 (15.3)	59.9 (10.3)	72.5 (15.9)
BMI, kg/m^2^	25.3 (4.0)	24.9 (2.9)	25.5 (4.5)
Education, n (%)			
<10 years	4 (10.3)	0 (0.0)	4 (15.4)
10 to 12 years	10 (25.6)	8 (61.5)	2 (7.7)
≥13 years	25 (64.1)	5 (38.5)	20 (76.9)
Marital status, n (%)			
Single	7 (17.9)	0 (0.0)	7 (26.9)
Married	30 (76.9)	12 (92.3)	18 (69.2)
Divorced or widowed	2 (5.1)	1 (7.7)	1 (3.8)
Living status, n (%)			
Lives alone	7 (17.9)	0 (0.0)	7 (26.9)
Lives with others	31 (79.5)	13 (100.0)	18 (69.2)
Occasionally lives with others	1 (2.6)	0 (0.0)	1 (3.8)
Employment status, n (%)			
Employed	29 (74.4)	8 (61.5)	21 (80.8)
Unemployed/retired	10 (25.6)	5 (38.5)	5 (19.2)
Current smoker, n (%)	4 (10.3)	0 (0.0)	4 (15.4)
Household income, JPY/year, n (%)			
<2 million	7 (17.9)	5 (38.5)	2 (7.7)
2–6 million	24 (61.5)	5 (38.5)	19 (73.1)
≥6 million	6 (15.4)	1 (7.7)	5 (19.2)
Unknown	2 (5.1)	2 (15.4)	0 (0.0)
Physical activity, MET-h/day	40.7 (4.2)	41.4 (3.9)	40.4 (4.4)
HbA1c, %	7.0 (0.8)	7.0 (0.5)	7.0 (0.9)
Fasting plasma glucose, mg/dL	129 (44)	117 (19)	135 (52)
eGFR, mL/min/1.73 m^2^	71(17)	73 (16)	70 (17)
Duration of diabetes, years	17.8 (10.1)	15.2 (9.5)	19.1 (10.3)
Diabetes treatment, n (%)			
Oral hypoglycemic agents	33 (84.6)	10 (76.9)	23 (88.5)
Sulphonylureas	5 (12.8)	2 (15.4)	3 (11.5)
Glinides	4 (10.3)	1 (7.7)	3 (11.5)
DPP-4 inhibitors	12 (30.8)	4 (30.8)	8 (30.8)
Biguanides	28 (71.8)	10 (76.9)	18 (69.2)
SGLT2 inhibitors	21 (53.8)	6 (46.2)	15 (57.7)
α-Glucosidase inhibitors	3 (7.7)	0 (0.0)	3 (11.5)
GLP-1 receptor agonist	8 (20.5)	4 (30.8)	4 (15.4)
Imeglimin	1 (2.6)	0 (0.0)	1 (3.8)
Insulin	19 (48.7)	6 (46.2)	13 (50.0)
Anti-hypertensive agents, n (%)	29 (74.4)	10 (76.9)	19 (73.1)
Lipid lowering drug, n (%)	25 (64.1)	9 (69.2)	16 (61.5)
Diuretics without loop diuretics, n (%)	1 (2.6)	0 (0.0)	1 (3.8)
J-DKT score, points	5.3 (1.4)	5.3 (1.3)	5.2 (1.4)
Energy intake, kcal/day	2061 (557)	1760 (335)	2211 (585)

Continuous variables are presented as mean ± standard deviation; categorical variables are presented as number (%). BMI, body mass index; MET, metabolic equivalents; DPP-4, dipeptidyl peptidase-4; eGFR, estimated glomerular filtration rate; GLP-1, glucagon-like peptide 1; J-DKT, Japanese version of diabetes knowledge test; SGLT2, sodium–glucose co-transporter-2.

**Table 2:** Mean daily protein and sodium, potassium, and phosphorus intakes and coefficient of variation, and within-to-between-individual variance ratio.

	Protein (g/day)			Sodium (mg/day)			Potassium (mg/day)			Phosphorus (mg/day)		
	24 h UC ^1^	DR ^2^	Difference ^3^	24 h UC ^1^	DR ^2^	Difference ^3^	24 h UC ^1^	DR ^2^	Difference ^3^	24 h UC ^1^	DR ^2^	Difference ^3^
Total (*n* = 39)												
Mean ^4^	77.8	77.0	0.8 (−4.7 to 6.3)	5315	4530	784 (312 to 1257) *	2796	2868	−72 (−314 to 171)	1060	1151	−91 (−194 to 12)
SD	23.0	23.0		2238	1648		995	917		376	346	
CV_w_ (%) ^5^	15.6	25.4	−9.8	24.0	27.9	−3.9	17.5	20.0	−2.5	17.4	24.1	−6.7
CV_b_ (%) ^6^	38.9	37.3	1.6	54.8	49.4	5.4	47.4	47.9	−0.5	47.3	39.7	7.6
VR ^7^	0.16	0.46	−0.30	0.19	0.32	−0.13	0.14	0.17	−0.03	0.14	0.37	−0.19
r ^8^	0.56 *			0.61 *			0.63 *			0.47 *		
Females (*n* = 13)												
Mean ^4^	65.9	69.5	−3.6 (−13.4 to 6.2)	4553	4052	501 (−191 to 1194)	2638	2769	−131 (−504 to 243)	968	1065	−97 (−242 to 47)
SD	23.8	18.5		1532	1342		846	775		329	263	
CV_w_ (%) ^5^	20.0	22.3	−2.3	27.9	23.0	5.0	19.3	16.0	3.3	17.8	20.9	−3.0
CV_b_ (%) ^6^	47.7	34.1	13.6	38.9	48.2	−9.4	41.7	43.9	−2.2	45.5	31.3	14.1
VR ^7^	0.18	0.43	−0.25	0.52	0.23	0.29	0.21	0.13	0.08	0.15	0.44	−0.26
r ^8^	0.83 *			0.48			0.75 *			0.49		
Males (*n* = 26)												
Mean ^4^	83.8	80.8	3.0 (−4.0 to 10.0)	5696	4769	927 (284 to 1570) *	2876	2918	−43 (−373 to 287)	1106	1194	−88 (−231 to 56)
SD	20.3	24.2		2443	1741		1061	981		392	375	
CV_w_ (%) ^5^	13.9	26.4	−12.5	22.4	29.4	−7.0	16.7	21.5	−4.8	17.2	25.2	−8.0
CV_b_ (%) ^6^	31.5	36.1	−4.6	56.8	48.1	8.7	49.9	50.2	−0.3	47.5	41.6	5.9
VR ^7^	0.19	0.53	−0.34	0.16	0.37	−0.21	0.11	0.18	−0.07	0.13	0.37	−0.19
r ^8^	0.39 *			0.58 *			0.58 *			0.33		

Notation: 24 h UC, 24 h urine collection; CV_b_, between-individual coefficient of variation; CV_w_, within-individual coefficient of variation; DR, dietary record; SD, standard deviation; VR, variance ratio. ^1^ Estimated by two non-consecutive days of 24 h UC. ^2^ Estimated by three non-consecutive days of DRs. ^3^ Calculated by (24 h UC-DR). ^4^ Difference values are shown as means (95% confidence interval). The values derived from 24 h UC were compared with those derived from the DR using a paired *t*-test. ^5^ Calculated by ([within-individual variance]^0.5^/mean) × 100. ^6^ Calculated by ([between-individual variance]^0.5^/mean) × 100. ^7^ Calculated by within-individual/between-individual variance ratio (σ_w_^2^/σ_b_^2^). ^8^ Values are expressed as Spearman correlation coefficients. * *p* < 0.05.

**Table 3:** Group size required to estimate the mean intake of protein and sodium, potassium, and phosphorus with 95% confidence intervals within a specified % deviation (D_0_) ^1^.

	Protein			Sodium			Potassium			Phosphorus		
D_0_	24 h UC ^2^	DR ^3^	Difference ^4^	24 h UC ^2^	DR ^3^	Difference ^4^	24 h UC ^2^	DR ^3^	Difference ^4^	24 h UC ^2^	DR ^3^	Difference ^4^
Total (*n* = 39)												
20%	17	20	−3	35	31	4	25	26	−1	25	21	4
10%	68	79	−11	138	124	14	99	104	−5	98	83	15
5%	271	313	−42	550	496	54	394	415	−21	391	331	60
2.5%	1081	1252	−171	2199	1981	218	1573	1659	−86	1561	1323	238
Females (*n* = 13)												
20%	26	16	10	23	28	−5	21	21	0	23	14	9
10%	103	64	39	89	110	−21	82	84	−2	92	55	37
5%	412	256	156	353	439	−86	325	336	−11	367	218	149
2.5%	1647	1022	625	1410	1756	−346	1298	1341	−43	1467	871	596
Males (*n* = 26)												
20%	12	20	−8	36	31	5	27	29	−2	25	23	2
10%	46	77	−31	144	122	22	107	115	−8	98	91	7
5%	183	308	−125	574	488	86	425	459	−34	392	363	29
2.5%	730	1231	−501	2294	1950	344	1699	1833	−134	1567	1452	115

Notation: 24 h UC, 24 h urine collection; CV_b_, between-individual coefficient of variation; CV_w_, within-individual coefficient of variation; DR, dietary record. ^1^ Group size was calculated using the following equation: G = 1.962 × [(CV_b_^2^ + CV_w_^2^)/D_0_^2^], where D_0_ = the specified % deviation of the group mean from group usual (“true”) mean intake. ^2^ Estimated by two non-consecutive days of 24 h UC. ^3^ Estimated by three non-consecutive days of DRs. ^4^ Calculated by (24 h UC-DR).

**Table 4:** Number of days required to ensure a specified correlation coefficient (r) between observed and usual (“true”) mean intake of protein and sodium, potassium, and phosphorus ^1^.

	Protein			Sodium			Potassium			Phosphorus		
r	24 h UC ^2^	DR ^3^	Difference ^4^	24 h UC ^2^	DR ^3^	Difference ^4^	24 h UC ^2^	DR ^3^	Difference ^4^	24 h UC ^2^	DR ^3^	Difference ^4^
Total (*n* = 39)												
0.75	1	1	0	1	1	0	1	1	0	1	1	0
0.8	1	1	0	1	1	0	1	1	0	1	1	0
0.9	1	2	−1	1	2	−1	1	1	0	1	2	−1
0.95	2	5	−3	2	3	−1	2	2	0	2	4	−2
Females (*n* = 13)												
0.75	1	1	0	1	1	0	1	1	0	1	1	0
0.8	1	1	0	1	1	0	1	1	0	1	1	0
0.9	1	2	−1	3	1	2	1	1	0	1	2	−1
0.95	2	4	−2	5	3	2	2	2	0	2	5	−3
Males (*n* = 26)												
0.75	1	1	0	1	1	0	1	1	0	1	1	0
0.8	1	1	0	1	1	0	1	1	0	1	1	0
0.9	1	3	−2	1	2	−1	1	1	0	1	2	−1
0.95	2	5	−3	2	4	−2	2	2	0	2	4	−2

Notation: 24 h UC, 24 h urine collection; DR, dietary record. ^1^ Number of days was calculated using the following equation: N_R_ = [r^2^/ (1 − r^2^)] × VR, where VR is the variance ratio as determined by σ_w_^2^/σ_b_^2^. In this analysis, r serves as a measure of confidence regarding the ranking or the classification of individuals in a population, based on the specific correlation coefficients. ^2^ Estimated by two non-consecutive days of 24 h UC. ^3^ Estimated by three non-consecutive days of DRs. ^4^ Calculated by (24 h UC-DR).

**Table 5:** Number of days required to estimate an individual’s mean intake of protein and sodium, potassium, and phosphorus with 95% confidence intervals within a specified % deviation (D_1_) ^1^.

	Protein			Sodium			Potassium			Phosphorus		
D_1_	24 h UC ^2^	DR ^3^	Difference ^4^	24 h UC ^2^	DR ^3^	Difference ^4^	24 h UC ^2^	DR ^3^	Difference ^4^	24 h UC ^2^	DR ^3^	Difference ^4^
Total (*n* = 39)												
30%	2	3	−1	3	4	−1	2	2	0	2	3	−1
20%	3	7	−4	6	8	−2	3	4	−1	3	6	−3
10%	10	25	−15	23	30	−7	12	16	−4	12	23	−11
5%	38	100	−62	89	120	−31	48	62	−14	47	89	−42
Females (*n* = 13)												
30%	2	3	−1	4	3	1	2	2	0	2	2	0
20%	4	5	−1	8	6	2	4	3	1	4	5	−1
10%	16	20	−4	30	21	9	15	10	5	13	17	−4
5%	62	77	−15	120	82	38	58	40	18	49	67	−18
Males (*n* = 26)												
30%	1	3	−2	3	4	−1	2	2	0	2	3	−1
20%	2	7	−5	5	9	−4	3	5	−2	3	7	−4
10%	8	27	−19	20	34	−14	11	18	−7	12	25	−13
5%	30	107	−77	78	133	−55	43	72	−29	46	98	−52

Notation: 24 h UC, 24 h urine collection; CV_w_, within-individual coefficient of variation; DR, dietary record. ^1^ Number of days was calculated using the following equation: N_I_ = (1.96 × CV_w_/D_1_)^2^, where D_1_ is used as the specific presentation. ^2^ Estimated by two non-consecutive days of 24 h UC. ^3^ Estimated by three non-consecutive days of DRs. ^4^ Calculated by (24 h UC-DR).

## Data Availability

The data presented in this study are available on request from the corresponding author. The data presented in this study are not publicly available because of privacy and ethical restrictions.

## Supplementary Materials

The following supporting information can be downloaded at: https://www.mdpi.com/article/10.3390/nu17111757/s1, Table S1: Simulation study of urinary excretion rates of protein, sodium, potassium, and phosphorus based on crude 24 h urinary excretion values and dietary record values; Table S2: Mean daily intakes of protein and sodium, potassium, and phosphorus along with their coefficients of variation and the within-to-between-individual variance ratios, after excluding individuals with incomplete 24 h urine collection based on the Joossens equation; Table S3: Mean daily intakes of protein and sodium, potassium, and phosphorus along with their coefficients of variation and within-to-between-individual variance ratios, after stratification by sub-group; Table S4: Mean daily urinary-phosphorus-excretion-to-urinary-nitrogen-excretion ratio (UP/UN), coefficient of variation, and within-to-between-individual variance ratio, and Spearman’s correlation coefficient between UP/UN and the phosphorus intake estimated by DRs; Table S5: Comparison of CV_w_, CV_b_, and variance ratio of protein, sodium, potassium, and phosphorus intakes across selected Japanese studies; Table S6: Estimated usual mean intake and variance ratios derived from Nagano Prefecture Health and Nutrition Surveys (2010, 2013, 2016, 2019, and 2022).

## Abbreviations

The following abbreviations are used in this manuscript:

24 h UC	24 h urine collection
BMI	Body mass index
CV_b_	Between-individual coefficient of variation
CV_w_	Within-individual coefficient of variation
DR	Dietary record
SD	Standard deviation
STFC-J	Standard Tables of Foods Composition in Japan
T2DM	Type 2 diabetes mellitus
VR	Variance ratio
