# Supplementary material for: Within- and Between-Individual Variations in Protein, Sodium, Potassium, and Phosphorus Intake Estimated from Urinary Biomarkers and Dietary Records in Individuals with Type 2 Diabetes Mellitus
Source: Nutrients. 2025 May 22;17(11):1757. doi: 10.3390/nu17111757 (PMC12158194; doi:10.3390/nu17111757)
Supplement: Supplementary file 1 [file nutrients-17-01757-s001.zip › nutrients-3628206-supplementary.pdf]

Supplemental Materials

"Within- and Between-Individual Variations in Protein, Sodium, Potassium, and Phosphorus Intake Estimated from Urinary Biomarkers and Dietary Records in Individuals with Type 2 Diabetes Mellitus"

Authors: Tomoya Takaoka, Daiki Watanabe, Manami Hosokawa, Kana Hosokawa, Satoshi Kubota, Yuko Kawai, Fumi Oono, Yumiko Inoue, Chieko Zakoji, Ako Oiwa, Ai Sato, Masanori Yamazaki, Mitsuhisa Komatsu

SUPPLEMENTAL TABLES

Table S1 Simulation study of urinary excretion rates of protein, sodium, potassium, and phosphorus based on crude 24 h urinary excretion values and dietary record values.

Table S2 Mean daily intakes of protein and sodium, potassium, and phosphorus along with their coefficients of variation and the within-to-between-individual variance ratios, after excluding individuals with incomplete 24 h urine collection based on the Joossens equation.

Table S3. Mean daily intakes of protein and sodium, potassium, and phosphorus along with their coefficients of variation and within-to-between-individual variance ratios, after stratification by sub-group.

Table S4 Mean daily urinary phosphorus excretion to urinary nitrogen excretion ratio (UP/UN), coefficient of variation, and within- to between-individual variance ratio, and Spearman's correlation coefficient between UP/UN and the phosphorus intake estimated by DRs.

Table S5 Comparison of CV_w, CV_b and variance ratio of protein, sodium, potassium, and phosphorus intake across selected Japanese studies.

Table S6 Estimated usual mean intake and variance ratios derived from Nagano Prefecture Health and Nutrition Surveys (2010, 2013, 2016, 2019, and 2022).

Within- and Between-Individual Variations in Protein, Sodium, Potassium, and Phosphorus Intake Estimated from Urinary Biomarkers and Dietary Records in Individuals with Type 2 Diabetes Mellitus. Tomoya Takaoka

Table S1. Simulation study of urinary excretion rates of protein, sodium, potassium, and phosphorus based on crude 24 h urinary excretion values and dietary record values.

	Protein (g/day)			Sodium (mg/day)			Potassium (mg/day)			Phosphorus (mg/day)		
	24-h UC[1]	DR[2]	Excretion rate (%)[3]	24-h UC[1]	DR[2]	Excretion rate (%)[3]	24-h UC[1]	DR[2]	Excretion rate (%)[3]	24-h UC[1]	DR[2]	Excretion rate (%)[3]
Total (n = 39)												
Mean[4]	54.0	77.0	70.2 (64.8 to 75.5)	4571	4530	102.0 (93.4 to 110.5)	2153	2868	76.3 (69.4 to 83.3)	665	1151	58.5 (52.2 to 64.9)
SD	16.0	23.0		1924	1648		766	917		236	346	
Females (n = 13)												
Mean[4]	45.4	69.5	64.8 (55.6 to 74.0)	3916	4052	100.8 (85.5 to 116.2)	2031	2769	74.2 (65.8 to 82.7)	607	1065	56.7 (47.8 to 65.6)
SD	16.4	18.5		1317	1342		651	775		207	263	
Males (n = 26)												
Mean[4]	58.0	80.8	72.8 (66.0 to 79.7)	4898	4769	102.6 (91.5 to 113.6)	2214	2918	77.4 (67.4 to 87.3)	694	1194	59.5 (50.7 to 68.3)
SD	14.0	24.2		2101	1741		817	981		246	375	

24-h UC, 24 h urine collection; DR, dietary record; SD, standard deviation.

[1] Estimated by two non-consecutive days of 24-h UC (values are crude data).

[2] Estimated by three non-consecutive days of DRs.

[3] Calculated by crude urinary excretion (assessed 24h-UC)/intake (assessed DR) *100.

[4] Excretion rates are shown mean (95% confidence interval).

Within- and Between-Individual Variations in Protein, Sodium, Potassium, and Phosphorus Intake Estimated from Urinary Biomarkers and Dietary Records in Individuals with Type 2 Diabetes Mellitus. Tomoya Takaoka

Table S2. Mean daily intakes of protein and sodium, potassium, and phosphorus along with their coefficients of variation and the within-to-between-individual variance ratios, after excluding individuals with incomplete 24 h urine collection based on the Joossens equation[1].

	Protein (g/day)			Sodium (mg/day)			Potassium (mg/day)			Phosphorus (mg/day)		
	24-h UC[2]	DR[3]	Difference[4]	24-h UC[2]	DR[3]	Difference[4]	24-h UC[2]	DR[3]	Difference[4]	24-h UC[2]	DR[3]	Difference[4]
Total (n = 20)												
Mean[5]	84.1	83.8	0.3 (-6.0 to 6.6)	5442	4570	872 (253 to 1492)*	3056	3210	-154 (-443 to 134)	1148	1261	-112 (-259 to 35)
SD	17.8	23.5		2027	1749		1003	962		376	363	
CV_w (%)[6]	11.9	24.6	-12.6	23.9	30.1	-6.2	15.3	19.4	-4.1	14.6	23.4	-8.8
CV_b (%)[7]	27.8	34.2	-6.5	47.4	51.4	-4.0	44.3	44.6	-0.3	44.5	37.8	6.7
VR[8]	0.18	0.52	-0.33	0.26	0.34	-0.09	0.12	0.19	-0.07	0.11	0.38	-0.28
r[9]	0.51*			0.58*			0.76*			0.43		
Females (n = 3)												
Mean[5]	74.3	72.4	1.9 (-18.0 to 22.2)	3499	3207	292 (-2133 to 2717)	3054	3047	7 (-1159 to 1174)	1168	1120	48 (-483 to 578)
SD	20.2	21.9		885	1558		917	569		377	254	
CV_w (%)[6]	7.9	22.3	-14.4	25.8	28.1	-2.3	5.7	5.9	-0.2	7.8	17.4	-9.7
CV_b (%)[7]	41.9	46.7	-4.8	24.4	84.1	-59.7	47.0	35.9	11.0	50.1	33.9	16.2
VR[8]	0.04	0.23	-0.19	1.12	0.11	1.01	0.01	0.03	-0.01	0.02	0.26	-0.24
r[9]	0.50			1.00			0.50			0.50		
Males (n = 17)												
Mean[5]	85.8	85.8	0.0 (-7.4 to 7.4)	5785	4811	974 (268 to 1681)*	3056	3239	-182 (-516 to 150)	1145	1285	-140 (-308 to 27)
SD	17.1	23.4		1983	1681		1030	1017		382	376	
CV_w (%)[6]	12.4	24.8	-12.4	23.5	30.0	-6.5	16.4	20.8	-4.3	15.5	24.0	-8.5
CV_b (%)[7]	25.6	31.9	-6.3	42.8	43.6	-0.8	45.3	46.5	-1.2	45.1	38.0	7.2
VR[8]	0.23	0.60	-0.37	0.30	0.47	-0.17	0.13	0.20	-0.07	0.12	0.40	-0.28
r[9]	0.45			0.44			0.74*			0.35		

Within- and Between-Individual Variations in Protein, Sodium, Potassium, and Phosphorus Intake Estimated from Urinary Biomarkers and Dietary Records in Individuals with Type 2 Diabetes Mellitus. Tomoya Takaoka

Notation: 24-h UC, 24 h urine collection; CV_b, between-individual coefficient of variation; CV_w, within-individual coefficient of variation; DR, dietary record; SGLT-2, sodium-glucose co-transporter-2; SD, standard deviation; VR, variance ratio. [1] The ratio of observed to expected creatinine excretion was calculated using the equations proposed by Joossens et al. [1, 2]. If the calculated ratio for a collection was less than 0.6 of the expected value, the UC was considered incomplete.

[2] Estimated by two non-consecutive days of 24-h UC.

[3] Estimated by three non-consecutive days of DRs.

[4] Calculated by (24-h UC - DR).

[5] Difference values are shown as the mean (95% confidence interval). The values derived from 24-h UC were compared with those derived from the DR using a paired *t*-test.

[6] Calculated by ([within-individual variance]$^{0.5}$/mean) ×100.

[7] Calculated by ([between-individual variance]$^{0.5}$/mean) ×100.

[8] Calculated by within-individual/between-individual VR (σ_w^2/σ_b^2).

[9] Values are expressed as Spearman correlation coefficients.

[*] $p<0.05$.

Within- and Between-Individual Variations in Protein, Sodium, Potassium, and Phosphorus Intake Estimated from Urinary Biomarkers and Dietary Records in Individuals with Type 2 Diabetes Mellitus. Tomoya Takaoka

Table S3. Mean daily intakes of protein and sodium, potassium, and phosphorus along with their coefficients of variation and within-to-between-individual variance ratios, after stratification by sub-group.

	Protein (g/day)			Sodium (mg/day)			Potassium (mg/day)			Phosphorus (mg/day)		
	24-h UC[1]	DR[2]	Difference[3]	24-h UC[1]	DR[2]	Difference[3]	24-h UC[1]	DR[2]	Difference[3]	24-h UC[1]	DR[2]	Difference[3]
Insulin use												
Yes (n = 19)												
Mean[4]	74.7	74.6	0.1 (-8.5 to 8.8)	5330	4515	816 (178 to 1454)*	2700	2792	-92 (-407 to 224)	996	1101	-105 (-294 to 83)
SD	22.2	23.3		1874	1771		1096	1031		407	344	
CV_w (%)[5]	11.7	28.5	-16.9	22.9	29.5	-6.6	19.9	20.6	-0.8	15.1	25.3	-10.2
CV_b (%)[6]	40.8	36.4	4.4	44.6	54.3	-9.7	54.5	57.8	-3.3	56.5	40.9	15.6
VR[7]	0.08	0.62	-0.53	0.26	0.29	-0.03	0.13	0.13	0.01	0.07	0.38	-0.31
r[8]	0.55*			0.59*			0.77*			0.31		
No (n = 20)												
Mean[4]	80.8	79.4	1.4 (-6.3 to 9.2)	5300	4545	756 (3 to 1508)*	2888	2941	-53 (-449 to 343)	1121	1198	-77 (-189 to 35)
SD	23.6	22.6		2560	1538		894	796		338	345	
CV_w (%)[5]	18.2	22.4	-4.2	25.0	26.3	-1.3	15.3	19.4	-4.2	18.9	23.0	-4.0
CV_b (%)[6]	37.5	38.2	-0.7	64.3	45.8	18.5	41.5	38.5	3.0	38.5	38.3	0.3
VR[7]	0.24	0.34	-0.11	0.15	0.33	-0.18	0.14	0.25	-0.12	0.24	0.36	-0.12
r[8]	0.51*			0.58*			0.44			0.62*		
SGLT2 inhibitor use												
Yes (n = 21)												
Mean[4]	81.7	79.2	2.5 (-6.3 to 11.3)	5333	4733	600 (-107 to 1307)	2834	2965	-131 (-467 to 205)	1130	1184	-53 (-209 to 102)
SD	22.5	24.5		1923	1563		856	966		368	382	
CV_w (%)[5]	13.9	26.8	-12.8	23.3	28.8	-5.5	16.7	21.8	-5.1	17.0	25.0	-8.0
CV_b (%)[6]	36.7	38.2	-1.5	45.8	40.6	5.2	39.7	47.9	-8.2	43.2	43.9	-0.7
VR[7]	0.14	0.49	-0.35	0.26	0.50	-0.24	0.18	0.21	-0.03	0.16	0.32	-0.17
r[8]	0.42			0.39			0.58*			0.31		

Within- and Between-Individual Variations in Protein, Sodium, Potassium, and Phosphorus Intake Estimated from Urinary Biomarkers and Dietary Records in Individuals with Type 2 Diabetes Mellitus. Tomoya Takaoka

No (n = 18)												
Mean[4]	73.3	74.5	-1.2 (-8.3 to 5.8)	5294	4294	1000 (329 to 1671)*	2752	2755	-3 (-390 to 384)	978	1113	-135 (-280 to 10)
SD	23.0	21.0		2585	1727		1147	851		374	297	
CV_w (%)[5]	17.8	23.5	-5.7	24.9	26.5	-1.7	18.5	17.3	1.2	17.9	22.7	-4.8
CV_b (%)[6]	41.2	36.2	4.9	65.2	59.6	5.6	56.7	48.4	8.3	51.6	33.6	18.0
VR[7]	0.19	0.42	-0.23	0.15	0.20	-0.05	0.11	0.13	-0.02	0.12	0.46	-0.34
r[8]	0.74*			0.74*			0.69*			0.75*		
Anti-hypertensive agent use												
Yes (n = 29)												
Mean[4]	73.0	73.2	-0.2 (-6.9 to 6.5)	5029	4442	587 (102 to 1072)*	2571	2729	-157 (-430 to 114)	1007	1083	-76 (-203 to 51)
SD	21.1	20.3		1745	1516		934	779		357	306	
CV_w (%)[5]	15.7	23.6	-7.9	20.6	27.2	-6.6	17.7	19.3	-1.6	15.8	22.9	-7.0
CV_b (%)[6]	38.0	34.8	3.2	44.8	45.2	-0.3	48.6	41.6	7.0	47.9	37.1	10.8
VR[7]	0.17	0.46	-0.29	0.21	0.36	-0.15	0.13	0.22	-0.08	0.11	0.38	-0.27
r[8]	0.52*			0.57*			0.57*			0.42*		
No (n = 10)												
Mean[4]	91.9	88.1	3.7 (-7.4 to 14.9)	6144	4785	1359 (38 to 2680)*	3450	3274	177 (-418 to 772)	1215	1349	-134 (-336 to 69)
SD	23.0	26.8		3191	1989		888	1155		396	383	
CV_w (%)[5]	15.2	28.4	-13.2	29.2	29.4	-0.2	16.8	21.1	-4.4	20.1	25.8	-5.7
CV_b (%)[6]	32.6	34.4	-1.8	68.9	60.4	8.5	32.9	54.9	-22.0	42.3	33.4	8.9
VR[7]	0.22	0.68	-0.46	0.18	0.24	-0.06	0.26	0.15	0.11	0.23	0.60	-0.37
r[8]	0.48			0.65*			0.54			0.62		
Household income (yen/year)												
<2 million (n = 10)												
Mean[4]	56.7	67.0	-10.3 (-19.5 to -1.1)*	4662	4257	405 (-536 to 1347)	2309	2585	-276 (-679 to 128)	683	1035	-353 (-645 to -60)*
SD	18.6	22.3		1425	1931		712	860		326	353	
CV_w (%)[5]	11.2	21.1	-9.8	18.4	26.2	-7.8	20.3	24.1	-3.8	21.5	26.3	-4.8
CV_b (%)[6]	46.8	51.7	-4.9	40.4	72.5	-32.1	39.8	48.3	-8.6	66.4	47.5	18.9

VR[7]	0.06	0.17	-0.11	0.21	0.13	0.08	0.26	0.25	0.01	0.11	0.31	-0.20
r[8]	0.86*			0.68			0.71			0.25		
2 to 6 million (n = 24)												
Mean[4]	82.6	78.9	3.7 (-3.5 to 11.0)	5514	4582	932 (270 to 1594)*	2850	2889	-39 (-415 to 337)	1145	1165	-20 (-152 to 112)
SD	21.4	24.0		2431	1654		990	979		359	373	
CV_w (%)[5]	15.9	26.8	-10.9	24.6	29.5	-4.9	15.7	20.9	-5.2	18.6	25.3	-6.7
CV_b (%)[6]	33.3	37.0	-3.7	57.8	47.0	10.8	47.0	51.3	-4.3	40.6	42.8	-2.2
VR[7]	0.23	0.53	-0.30	0.18	0.39	-0.21	0.11	0.17	-0.05	0.21	0.35	-0.14
r[8]	0.34			0.53*			0.51*			0.44		
≥6 million (n = 6)												
Mean[4]	80.9	81.3	-0.4 (-13.8 to 12.9)	5255	4428	827 (-662 to 2116)	3110	3106	4 (-415 to 423)	1149	1220	-71 (-283 to141)
SD	14.6	20.2		2259	1501		1193	809		249	235	
CV_w (%)[5]	13.2	25.2	-12.0	28.2	26.5	1.6	19.8	13.2	6.6	8.0	18.0	-10.0
CV_b (%)[6]	22.5	24.1	-1.5	55.8	47.1	8.7	52.6	43.5	9.1	30.9	21.9	8.9
VR[7]	0.34	1.09	-0.75	0.26	0.32	-0.06	0.14	0.09	0.05	0.07	0.67	-0.61
r[8]	0.43			0.77			1.00*			0.54		
Unknown (n = 2)												
Mean[4]	85.1	77.2	7.9 (-367.0 to 382.8)	5397	5173	224 (-21305 to 21754)	2915	2896	19 (-8140 to 8178)	1095	1180	-86 (-3980 to 3809)
SD	38.8	10.7		2410	847		1040	420		322	198	
CV_w (%)[5]	21.3	15.3	6.0	13.9	16.6	-2.7	20.7	14.9	5.8	13.1	18.1	-5.0
CV_b (%)[6]	73.0	5.0	68.0	74.8	15.3	59.5	54.4	12.7	41.6	47.5	9.7	37.8
VR[7]	0.09	9.36	-9.28	0.03	1.17	-1.14	0.15	1.37	-1.22	0.08	3.49	-3.41
r[8]	1.00			1.00			1.00			-1.00		

24-h UC, 24 h urine collection; CV_b, between-individual coefficient of variation; CV_w, within-individual coefficient of variation; DR, dietary record; SD, standard deviation; SGLT2, sodium-glucose co-transporter-2; VR, variance ratio.

[1] Estimated by two non-consecutive days of 24-h UC.

[2] Estimated by three non-consecutive days of DRs.

Within- and Between-Individual Variations in Protein, Sodium, Potassium, and Phosphorus Intake Estimated from Urinary Biomarkers and Dietary Records in Individuals with Type 2 Diabetes Mellitus. Tomoya Takaoka

[3] Calculated by (24-h UC - DR).

[4] Difference values are shown as the mean (95% confidence interval). The values derived from 24-h UC were compared with those derived from the DR using the paired *t*-test.

[5] Calculated by ([within-individual variance]$^{0.5}$/mean) ×100.

[6] Calculated by ([between-individual variance]$^{0.5}$/mean) ×100.

[7] Calculated by the within-individual/between-individual VR (σ_w^2/σ_b^2).

[8] Values are expressed as Spearman correlation coefficients.

[*] $P < 0.05$.

Within- and Between-Individual Variations in Protein, Sodium, Potassium, and Phosphorus Intake Estimated from Urinary Biomarkers and Dietary Records in Individuals with Type 2 Diabetes Mellitus. Tomoya Takaoka

Table S4. Mean daily urinary phosphorus excretion to urinary nitrogen excretion ratio (UP/UN), coefficient of variation, and within- to between-individual variance ratio, and Spearman's correlation coefficient between UP/UN and the phosphorus intake estimated by DRs.

	UP/UN (mg/g)[1]	Phosphorus (mg/day)[2]
	Total (n = 39)	
Mean	78.4	1151
SD	20.9	346
CV_w (%)[3]	17.2	24.1
CV_b (%)[4]	33.8	39.7
VR[5]	0.26	0.37
r[6]	0.09	
	Females (n = 13)	
Mean	84.2	1065
SD	13.4	263
CV_w (%)[3]	11.1	20.9
CV_b (%)[4]	19.8	31.3
VR[5]	0.32	0.44
r[6]	0.16	
	Males (n = 26)	
Mean	75.5	1194
SD	23.4	375
CV_w (%)[3]	20.0	25.2
CV_b (%)[4]	39.3	41.6
VR[5]	0.26	0.37
r[6]	0.12	

24-h UC, 24 h urine collection; CV_b, between-individual coefficient of variation; CV_w, within-individual coefficient of variation; DR, dietary record; SD, standard deviation; VR, variance ratio.

[1] Calculated by (urinary phosphorus excretion/urinary nitrogen excretion). Urinary phosphorous and nitrogen estimated by two non-consecutive days of 24-h UC.

[2] Estimated by three non-consecutive days of DRs.

[3] Calculated by ([within-individual variance]$^{0.5}$/mean) ×100.

[4] Calculated by ([between-individual variance]$^{0.5}$/mean) ×100.

[5] Calculated by within-individual/between-individual variance ratio (σ_w^2/σ_b^2).

[6] Values are expressed as Spearman correlation coefficients.

Within- and Between-Individual Variations in Protein, Sodium, Potassium, and Phosphorus Intake Estimated from Urinary Biomarkers and Dietary Records in Individuals with Type 2 Diabetes Mellitus. Tomoya Takaoka

Table S5. Comparison of CV_w, CV_b and variance ratio of protein, sodium, potassium, and phosphorus intake across selected Japanese studies[1].

	This study		Ogawa, 1999		Tokudome, 2002	Fukumoto, 2013				Tsubota-Utsugi, 2013		Taguchi, 2017	Watanabe, 2018		Watanabe, 2019		Yoshizawa, 2019	Suzuki, 2024	
Sex	F	M	F[2]	M[3]	F[4]	F[5]	F[6]	M[7]	M[8]	F[9]	M[10]	M[11]	F[12]	M[13]	F[14]	M[15]	FM[p6]	F[17]	M[18]
Protein																			
DR																			
CV_w (%)	22.3	26.4	23.6	23.1	19.4	25.5	23.5	25.4	23.7	23.4	22.6	30.7			17.9	19.5	22		
CV_b (%)	34.1	36.1	15.0	17.0	13.1	16.6	13.4	19.8	14.5	14.4	15.5	12.0			14.9	17.0			
VR	0.43	0.53	2.5	1.8	2.2	2.37	3.08	1.64	2.67	2.6	2.1	6.53			1.2	1.15	1.2		
24-h UC																			
CV_w (%)	20.0	13.9											1.83	1.57					
CV_b (%)	47.7	31.5											2.26	1.80					
VR	0.18	0.19											0.81	0.87					
Sodium																			
DR																			
CV_w (%)	23.0	29.4	28.5	28.7		33.7	34.4	35.7	34.1	28.2	28.0	29.8					31	28.7	28.7
CV_b (%)	48.2	48.1	16.1	19.7		17.7	15.9	20.2	14.7	16.9	16.7	17.3						22.4	23.1
VR	0.23	0.37	3.1	2.1		3.61	4.67	3.13	5.35	2.8	2.8	2.95					1.9		
24-h UC																			
CV_w (%)	27.9	22.4											5.33	4.97				29.5	31.0
CV_b (%)	38.9	56.8											10.31	7.95				22.2	23.0
VR	0.52	0.16											0.52	0.62					
Potassium																			
DR																			
CV_w (%)	16.0	21.5	23.6	27.3	20.4	27.4	26.7	26.0	23.9	24.2	26.5	26.5					23	24.8	23.3
CV_b (%)	43.9	50.2	17.4	18.3	17.7	21.3	17.0	23.8	16.8	16.7	17.5	20.6						28.8	22.9
VR	0.13	0.18	1.8	2.2	1.3	1.66	2.46	1.19	2.03	2.1	2.3	1.64					0.8		
24-h UC																			
CV_w (%)	19.3	16.7																25.2	25.3
CV_b (%)	41.7	49.9																27.0	27.9
VR	0.21	0.11																	
Phosphorus																			
DR																			
CV_w (%)	20.9	25.2	22.9	22.6	19.3	24.6	22.4	24.0	22.7	22.8	22.5	28.3					22		
CV_b (%)	31.3	41.6	15.8	17.7	14.5	19.1	15.9	22.4	15.7	14.6	16.5	16.0							

VR	0.44	0.37	2.1	1.6	1.8	1.65	1.98	1.15	2.10	2.4	1.9	3.15		1.0
24-h UC														
CV_w (%)	17.8	17.2												
CV_b (%)	45.5	47.5												
VR	0.15	0.13												

24-h UC, 24 h urine collection; CV_b, between-individual coefficient of variation; CV_w, within-individual coefficient of variation; DR, dietary record; SD, standard deviation; F, females; M, males; VR, variance ratio.

[1] The CV_w, CV_b, and VR were derived from each study.

[2] Ogawa et al. [3] included 60 females with a mean age of 61.2 years who completed 3 to 12-day DRs.

[3] Ogawa et al. [3] included 59 males with a mean age of 62.5 years who completed 3 to 12-day DRs.

[4] Tokudome et al. [4] included 80 female dietitians with a mean age of 48 years who completed 28-day DRs.

[5] Fukumoto et al. [5] included 58 females with a mean age of 39.0 years who completed 16-day DRs.

[6] Fukumoto et al. [5] included 63 females with a mean age of 58.9 years who completed 16-day DRs.

[7] Fukumoto et al. [5] included 54 males with a mean age of 40.5 years who completed 16-day DRs.

[8] Fukumoto et al. [5] included 67 males with a mean age of 61.5 years who completed 16-day DRs.

[9] Tsubota-Utsugi et al. [6] included 58 females with a mean age of 61 years who completed 12-day DRs.

[10] Tsubota-Utsugi et al. [6] included 55 males with a mean age of 62 years who completed 12-day DRs.

[11] Taguchi et al. [7] included 56 males with a mean age of 37.9 years who completed 7-day DRs.

[12] Watanabe et al. [8] included 204 females with stage G3, G4, or G5 who were not undergoing dialysis for chronic kidney disease who completed 2-day 24-h UC.

[13] Watanabe et al. [8] included 86 males with stage G3, G4, or G5 who were not undergoing dialysis for chronic kidney disease who completed 2-day 24-h UC.

[14] Watanabe et al. [9] included 65 females with a mean age of 72.5 years who completed 7-day DRs.

[15] Watanabe et al. [9] included 78 males with a mean age of 73.8 years who completed 7-day DRs.

[16] Yoshizawa et al. [10] included 33 men and 98 women with a mean age of 58 years who completed 12-day DRs.

[17] Suzuki et al. [11] included 122 females with a mean age of 58 years who completed 12-day DRs and 5-day 24-h UC.

Within- and Between-Individual Variations in Protein, Sodium, Potassium, and Phosphorus Intake Estimated from Urinary Biomarkers and Dietary Records in Individuals with Type 2 Diabetes Mellitus. Tomoya Takaoka

[18] Suzuki et al. [11] included 80 males with a mean age of 59 years who completed 12-day DRs and 5-day 24-h UC.

Within- and Between-Individual Variations in Protein, Sodium, Potassium, and Phosphorus Intake Estimated from Urinary Biomarkers and Dietary Records in Individuals with Type 2 Diabetes Mellitus. Tomoya Takaoka

Table S6. Estimated usual mean intake and variance ratios derived from Nagano Prefecture Health and Nutrition Surveys (2010, 2013, 2016, 2019, and 2022)[1].

	Protein (g/day)			Sodium (mg/day)[2]			Potassium (mg/day)			Phosphorus (mg/day)		
	Mean[3]	SD	VR[4,5]	Mean[3]	SD	VR[4,5]	Mean[3]	SD	VR[4,5]	Mean[3]	SD	VR[4,5]
Females												
2010 (n = 537)	65.5	13.6	1.58	4173	945	1.39	2378	568	1.14	972	196	1.38
2013 (n = 669)	64.9	13.7	0.88	3976	1024	0.99	2343	626	0.60	960	222	0.72
2016 (n = 647)	63.7	13.6	0.90	3819	906	1.44	2343	766	0.42	943	231	0.69
2019 (n = 428)	65.4	16.0	0.68	3780	984	1.01	2370	697	0.70	958	262	0.52
2022 (n = 560)	68.3	14.0	1.25	3740	1024	0.79	2424	739	0.52	993	242	0.76
Weighted average[6]	65.5			3902			2370			965		
Males												
2010 (n = 484)	78.2	17.0	0.77	4843	1142	0.85	2553	732	0.44	1136	268	0.62
2013 (n = 581)	77.7	16.8	1.02	4528	1181	0.93	2492	762	0.44	1107	260	0.79
2016 (n = 575)	76.0	17.9	0.67	4449	1102	1.24	2525	860	0.29	1081	305	0.34
2019 (n = 349)	80.3	20.1	0.62	4528	1181	0.91	2576	644	1.01	1128	288	0.68
2022 (n = 484)	81.8	17.2	1.15	4370	1024	1.26	2644	788	0.49	1142	275	0.81
Weighted average[6]	78.6			4540			2553			1116		

DR, dietary record; n, number of individuals; SD, standard deviation; VR, variance ratio.

[1] Data were derived from Nagano Prefecture Health and Nutrition Surveys[12-16]. The individuals were adults aged ≥18 years. Dietary intake data were collected using a one-day semi-weighted household DR.

[2] Calculated as salt (g/day)/2.54 × 1000.

[3] Usual mean intakes were reported by adopting the best-power method [17] to a one-day semi-weighted household DR in all individuals.

[4] VR as determined by σ_w^2/σ_b^2.

[5] VR was reported using a two-day DR sub-sample; however, the population numbers were unavailable for each year.

[6] Weighted average was calculated by multiplying the weight associated with the value of each study by the number of participants.

Within- and Between-Individual Variations in Protein, Sodium, Potassium, and Phosphorus Intake Estimated from Urinary Biomarkers and Dietary Records in Individuals with Type 2 Diabetes Mellitus. Tomoya Takaoka

References

[1] Murakami K, Sasaki S, Takahashi Y, Uenishi K, Watanabe T, Kohri T et al. Sensitivity and specificity of published strategies using urinary creatinine to identify incomplete 24-h urine collection. Nutrition. 2008;24:16-22. https://doi.org/10.1016/j.nut.2007.09.001.

[2] Knuiman J T, Hautvast J G, van der Heyden L, Geboers J, Joossens J V, Tornqvist H et al. A multi-centre study on completeness of urine collection in 11 European centres. I. Some problems with the use of creatinine and 4-aminobenzoic acid as markers of the completeness of collection. Hum Nutr Clin Nutr. 1986;40:229-37.

[3] Ogawa K, Tsubono Y, Nishino Y, Watanabe Y, Ohkubo T, Watanabe T et al. Inter- and intra-individual variation of food and nutrient consumption in a rural Japanese population. Eur J Clin Nutr. 1999;53:781-5. https://doi.org/10.1038/sj.ejcn.1600845.

[4] Tokudome Y, Imaeda N, Nagaya T, Ikeda M, Fujiwara N, Sato J et al. Daily, weekly, seasonal, within- and between-individual variation in nutrient intake according to four season consecutive 7 day weighed diet records in Japanese female dietitians. J Epidemiol. 2002;12:85-92. https://doi.org/10.2188/jea.12.85.

[5] Fukumoto A, Asakura K, Murakami K, Sasaki S, Okubo H, Hirota N et al. Within- and between-individual variation in energy and nutrient intake in Japanese adults: effect of age and sex differences on group size and number of records required for adequate dietary assessment. J Epidemiol. 2013;23:178-86. https://doi.org/10.2188/jea.je20120106.

[6] Tsubota-Utsugi M, Imai E, Nakade M, Matsumoto T, Tsuboyama-Kasaoka N, Nishi N et al. Evaluation of the prevalence of iodine intakes above the tolerable upper intake level from four 3-day dietary records in a Japanese population. J Nutr Sci Vitaminol (Tokyo). 2013;59:310-6. https://doi.org/10.3177/jnsv.59.310.

[7] Taguchi C, Kishimoto Y, Fukushima Y, Saita E, Tanaka M, Takahashi Y et al. Dietary Polyphenol Intake Estimated by 7-Day Dietary Records among Japanese Male Workers: Evaluation of the Within- and Between-Individual Variation. J Nutr Sci Vitaminol (Tokyo). 2017;63:180-5. https://doi.org/10.3177/jnsv.63.180.

[8] Watanabe D, Machida S, Matsumoto N, Shibagaki Y, Sakurada T. Age Modifies the Association of Dietary Protein Intake with All-Cause Mortality in Patients with Chronic Kidney Disease. Nutrients. 2018;10. https://doi.org/10.3390/nu10111744.

[9] Watanabe D, Nanri H, Yoshida T, Yamaguchi M, Sugita M, Nozawa Y et al. Validation of Energy and Nutrition Intake in Japanese Elderly Individuals

Estimated Based on a Short Food Frequency Questionnaire Compared against a 7-day Dietary Record: The Kyoto-Kameoka Study. Nutrients. 2019;11. https://doi.org/10.3390/nu11030688.

[10] Yoshizawa K, Willett W C, Yuan C. Reliability of Repeated Measures of Nutrient Intake by Diet Records in Residents in the Western Region of Japan. Nutrients. 2019;11. https://doi.org/10.3390/nu11102515.

[11] Suzuki A, Takachi R, Ishihara J, Maruya S, Ishii Y, Kito K et al. Urinary Biomarkers in Screening for the Usual Intake of Fruit and Vegetables, and Sodium, Potassium, and the Sodium-to-Potassium Ratio: Required Number and Accuracy of Measurements. Nutrients. 2024;16. https://doi.org/10.3390/nu16030442.

[12] The Health and Welfare Department health promotion department Nagano prefecture. Inhabitant of a prefecture health and nutritional investigation. 2010. (Accessed January 5 2025 at https://www.pref.nagano.lg.jp/kenko-choju/kenko/kenko/kenko/chosa/chousa22.html.)

[13] The Health and Welfare Department health promotion department Nagano prefecture. Inhabitant of a prefecture health and nutritional investigation. 2013. (Accessed January 5 2025 at https://www.pref.nagano.lg.jp/kenko-choju/kenko/kenko/kenko/chosa/chousa25-2.html.)

[14] The Health and Welfare Department health promotion department Nagano prefecture. Inhabitant of a prefecture health and nutritional investigation. 2016. (Accessed January 5 2025 at https://www.pref.nagano.lg.jp/kenko-choju/kenko/kenko/kenko/chosa/chousa28.html.)

[15] The Health and Welfare Department health promotion department Nagano prefecture. Inhabitant of a prefecture health and nutritional investigation. 2019. (Accessed January 5 2025 at https://www.pref.nagano.lg.jp/kenko-choju/kenko/kenko/kenko/chosa/chousa-r1.html.)

[16] The Health and Welfare Department health promotion department Nagano prefecture. Inhabitant of a prefecture health and nutritional investigation. 2022. (Accessed January 5 2025 at https://www.pref.nagano.lg.jp/kenko-choju/kenko/kenko/kenko/chosa/chousar4.html.)

[17] Nusser S M, Carriquiry A L, Dodd K W, Fuller W A. A semiparametric transformation approach to estimating usual daily intake distributions. J Am Stat Assoc. 1996;91:1440-9. https://doi.org/Doi 10.2307/2291570.
